# Severe thrombocytopenia induced by tirofiban after percutaneous coronary intervention: a case report

**DOI:** 10.1186/s13256-023-04169-5

**Published:** 2023-10-15

**Authors:** Ze-Mu Wang, Bin Wang, Ya-Fei Li, Bei Chen, Qin Shen, Dian-Fu Li, Lian-Sheng Wang

**Affiliations:** 1 Department of Cardiology, The First Affiliated Hospital of Nanjing Medical University, Nanjing, China; 2 Institute of Nephrology, Zhong Da Hospital, Southeast University School of Medicine, Nanjing, China; 3 Department of Cardiology, The Affiliated Suzhou Hospital of Nanjing Medical University, Suzhou Municipal Hospital, Gusu School, Nanjing Medical University, Suzhou, China

**Keywords:** Glycoprotein IIb/IIIa receptor antagonists, Tirofiban, Acute coronary syndromes, Percutaneous coronary intervention

## Abstract

**Background:**

Tirofiban is a nonpeptide glycoprotein IIb/IIIa receptor antagonist used widely in patients subjected to percutaneous coronary intervention. While the usage of tirofiban sets an important clinical benefit, severe thrombocytopenia can occur with use of this agent.

**Case presentation:**

A 76-year-old Chinese man was admitted with 1-month history of sudden onset of chest tightness. He was diagnosed as having subacute inferior myocardial infarction, and percutaneous coronary intervention was performed. After the procedure, patient received tirofiban at 0.15 µg/kg/minute for 4 h. A blood sample was obtained for a complete blood count; severe thrombocytopenia was reported according to routine orders at our hospital. All antiplatelet drugs including tirofiban, aspirin, and clopidogrel were immediately discontinued. The patient received platelet transfusions and was treated with immunoglobulin G. Two days later, the patient’s platelet count had increased to 75 × 10^9^/L. There was a significant improvement after day 5, and the platelet count was 112 × 10^9^/L. Seven days after the acute thrombocytopenia, he was discharged with normal platelet count.

**Conclusions:**

Clinicians should be particularly aware of tirofiban-induced thrombocytopenia in routine practice.

## Background

Glycoprotein IIb/IIIa receptor antagonists (GPRAs), including abciximab, eptifibatide, and tirofiban, are widely used in the treatment of patients with acute coronary syndromes (ACS) [1]. These agents have been extensively studied in several randomized trials, which have demonstrated that the use of GPRAs may reduce the incidence of myocardial infarction (MI) and composite cardiac outcomes in patients subjected to percutaneous coronary intervention (PCI) [2]. However, severe thrombocytopenia can occur with use of these agents [3]. Drug-induced thrombocytopenia may result from a number of diverse etiologies; it is important for clinicians to make an accurate diagnosis to guide treatment decisions and to inform prognosis. Here, we report a case of severe thrombocytopenia within 4 hours of tirofiban administration after PCI for subacute inferior MI.

## Case presentation

**Fig. 1:**
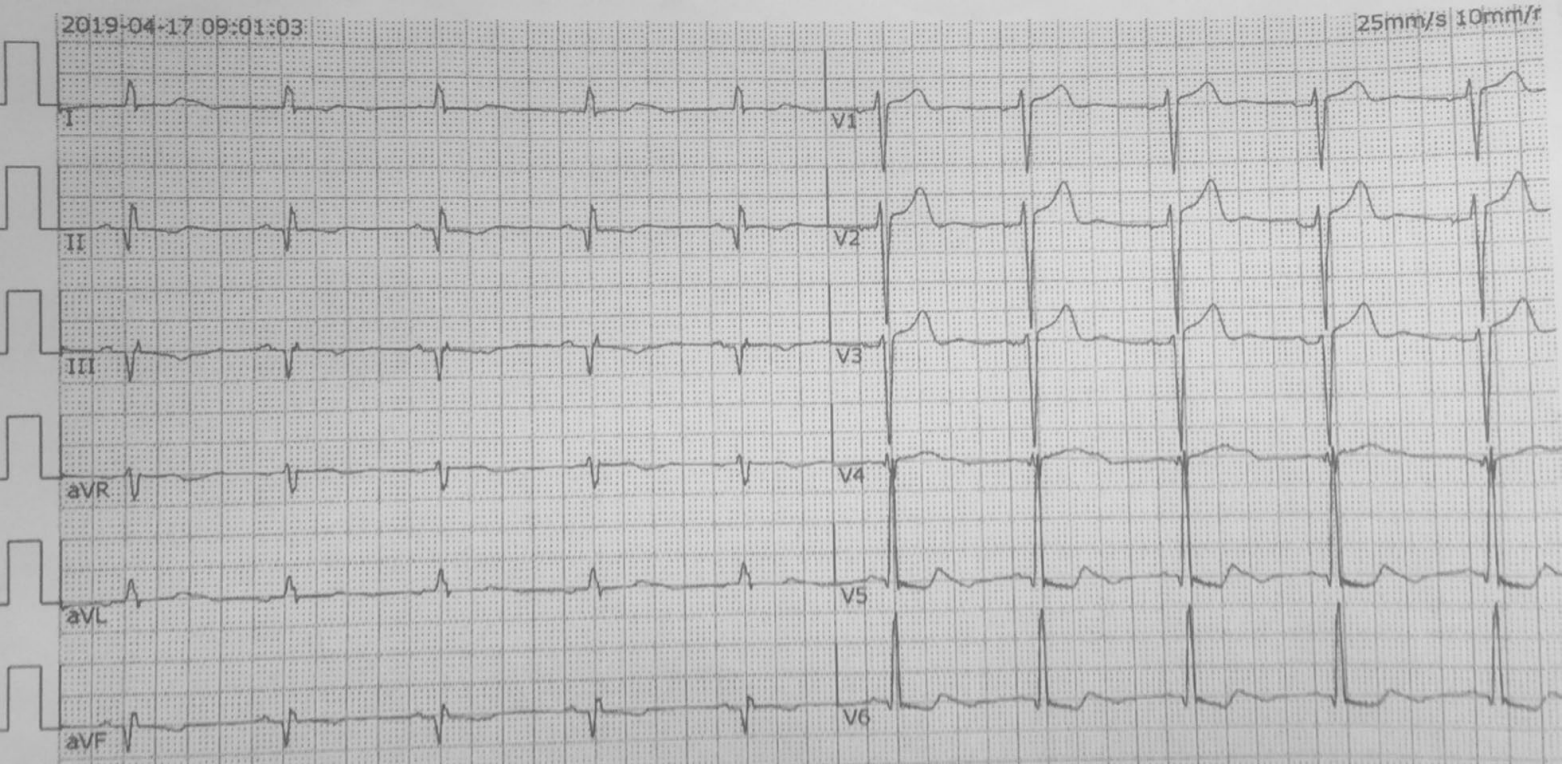
Initial electrocardiogram of a 76-year-old man admitted with 1-month history of chest tightness

**Fig. 2:**
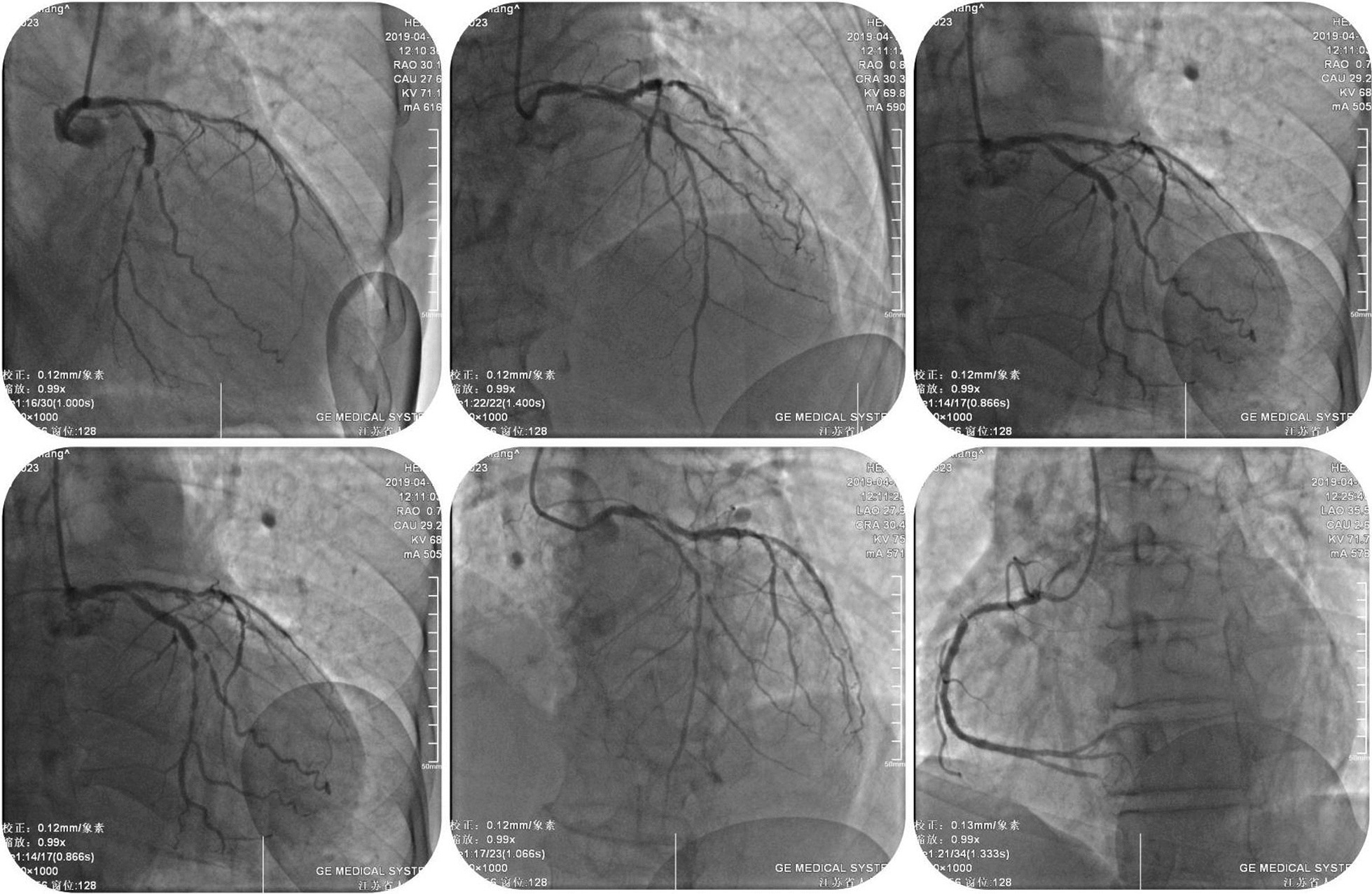
Coronary angiography revealed an evidence of left main and three vessel coronary artery disease

**Fig. 3:**
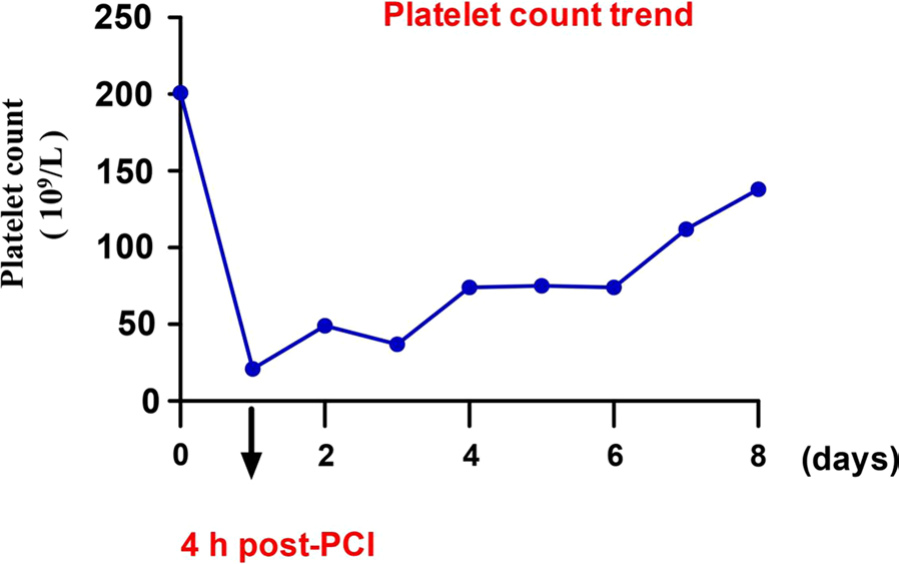
Platelet count trend during hospitalization

A 76-year-old Chinese man was admitted with 1-month history of sudden onset of chest tightness. A month previously, the symptom started while he was doing exercise. It was associated with diaphoresis and shortness of breath, without chest pain. The resting 12-lead electrocardiogram (ECG) showed abnormal Q waves in leads II, III, and aVF, and the cardiac troponin T was elevated. He was diagnosed as having ACS and was treated at a local hospital. Owing to the fact that the hospital was unable to perform coronary angiography (CAG), the patient was discharged home several days later on aspirin (100 mg qd), clopidogrel (75 mg qd), metoprolol (12.5 mg qd), and rosuvastatin (10 mg qn). On admission to our hospital, the patient still had a mildly elevated level of high-sensitivity cardiac troponin T (21.58 ng/L). His initial ECG showed abnormal Q waves in the inferior (II, III, and avF) leads (Fig. 1), which may indicate subacute inferior MI. His routine blood test was normal, including a platelet count of 201 × 10^9^/L and a white blood cell count of 5.86 × 10^9^/L. He had no history of blood dyscrasia and denied any history of smoking or drinking. The patient underwent CAG in our hospital after admission. CAG revealed evidence of left main (LM) and three-vessel coronary artery disease (Fig. 2). Low-dose heparin (2000 units) was given during the procedure. We consider the optimal revascularization technique for this patient to be coronary artery bypass graft (CABG) surgery and did not perform PCI. However, the patient decide to be treated with PCI rather than CABG. Four days later, CAG was performed again; multivessel coronary intervention was performed with drug-eluting stents and drug-coated balloon. The procedure lasted for about 2 hours, and the dose of heparin given was 6500 units. After the procedure, the patient was transferred in stable condition to the ward and treated by intravenous tirofiban at 0.15 µg/kg/minute. Post-PCI medications included aspirin 100 mg qd, clopidogrel 75 mg qd, metoprolol succinate 12.5 mg qd, rosuvastatin 10 mg qn, and benazepril 5 mg qd. A blood sample was obtained for a complete blood count 4 hours after the procedure, according to routine orders at our hospital. His platelet count was 21 × 10^9^/L, which was confirmed by manual examination of the blood film. The patient’s hemoglobin level was 119 g/L. Tirofiban infusion was stopped by 4 hours, and other antiplatelet drugs including aspirin and clopidogrel were immediately discontinued. A heparin-induced thrombocytopenia (HIT) platelet factor 4 antibody test was performed, and the result was negative [4]. Over the next 12 hours, the patient received 10 unit platelet transfusions to prevent hemorrhage, and his platelet count had increased to 49 × 10^9^/L. Another analysis completed later indicated that his platelet count was 37 × 10^9^/L. Additionally, immunoglobulin G (10g) was given. Aspirin and clopidogrel were resumed the next day, and the patient received another 10 unit platelet transfusions. Two days later, the patient’s platelet count had increased to 75 × 10^9^/L. The course of the patient’s platelet count is shown in Fig. 3. There was a significant improvement after day 5, and the platelet count was 112 × 10^9^/L. Seven days after the acute profound thrombocytopenia, his platelet count was 138 × 10^9^/L, and he was discharged with no hemorrhagic sequelae.

## Discussion and conclusions

Tirofiban is a nonpeptide glycoprotein (GP) IIb/IIIa receptor antagonist used in patients subjected to PCI for the prevention of acute stent thrombosis and reduction of major adverse coronary events [5]. It inhibits platelet aggregation by preventing the attachment of fibrinogen and von Willebrand factor to the GP IIb/IIIa receptor on the thrombocyte surface [6]. While the usage of GP IIb/IIIa inhibitors sets an important clinical benefit, the reported incidence of thrombocytopenia induced by tirofiban ranges from 0.4% to 5.6% [7]. Thus, clinicians should be particularly aware of tirofiban-induced thrombocytopenia in routine practice.

Besides tirofiban, aspirin and clopidogrel are widely used as antiplatelet agents; they have also been reported to be associated with thrombocytopenia [8]. However, the described patient had used these two drugs for 1 month before admission, and his routine blood test showed a normal platelet count after admission. Therefore, the thrombocytopenia was not caused by the dual antiplatelet therapy.

The most well-known medication that can induce thrombocytopenia is heparin [9]. HIT is the most important complication of heparin therapy during PCI in cardiac patients. There are two types of HIT. Type I HIT is a transient, mild drop in platelet counts 48–72 hours after initiation of heparin therapy. It occurs because of direct heparin-induced platelet aggregation and is usually clinically harmless [7]. Type II HIT is an adverse immune-mediated reaction due to antibodies formed against heparin–platelet factor 4 complexes, which is usually associated with thrombosis risk. It is more severe than type I HIT and should be suspected when patients show a reduction in the platelet count to less than 100,000 per cubic millimeter or more than 50% of the baseline value 5–15 days after initiation of heparin therapy [10, 11]. The 4T's score (the severity of Thrombocytopenia, its Timing of heparin exposure, appearance of new Thrombosis, and differential diagnosis by exclusion of oTher causes) has been utilized as a clinical assessment tool to evaluate the likelihood of HIT [11, 12]. Our patient’s 4T's score was 3 points. Thus, the suspicion for HIT was low. In addition, we carried out an immunoassay to examine the presence of HIT antibodies; the negative result indicated that our patient did not have an HIT type II reaction. It is important to exclude HIT and other causes of thrombocytopenia to diagnose tirofiban-induced thrombocytopenia accurately and treat patients appropriately.

A recent study based on pre-procedural characteristics for early prediction of thrombocytopenia before patients were exposed to tirofiban has developed a simple risk model to predict thrombocytopenia associated with periprocedural tirofiban exposure [7]. Five independent risk factors, including age ≥ 65 years (2 points), white blood cell ≥ 12 × 10^9^ /L (1 point), diabetes mellitus (2 points), congestive heart failure (2 points), and chronic kidney disease (1 point), were identified as risk factors in the scoring system. According to the scoring system, ≥ 7 points, 3–6 points, and ≤ 2 points indicate high risk, moderate risk and low risk. For our patient, this score only indicates a moderate risk (4 points), calculated based on age and congestive heart failure. Therefore, a further predictive model is still needed to help doctors identify high-risk patients in clinical practice [7].

The efficacy of tirofiban for patients with MI who undergo PCI was positively correlated with its dose; high dose can enhance the clinical effects, but also increase the hemorrhagic risk [13]. The appropriate dose could be adopted by reference to the specific conditions of patients under assessment of bleeding risk, and a common recommended clinical dose of 10 µg/kg may be appropriate for patients without high hemorrhagic risk, followed by continuous intravenous injection at 0.15 µg/kg/minute [13]. It is important to monitor platelet counts closely after initiation of tirofiban infusion [14]. For these patients, testing platelet counts before treatment, 2–4 h following the start of infusion, and at 24 h would detect most cases of acute thrombocytopenia [1, 15]. Discontinuation of tirofiban is usually sufficient for treatment of thrombocytopenia because it is cleared from the circulation within the first hours of cessation of the drug [14, 16].

In conclusion, this report demonstrates an example of acute severe thrombocytopenia induced by tirofiban and endorses the importance of platelet count monitoring after initiating therapy with this agent in clinical practice.

## Data Availability

Not applicable.

## Abbreviations

ACS: Acute coronary syndromes
CABG: Coronary artery bypass graft
CAG: Coronary angiography
ECG: Electrocardiogram
GP: Glycoprotein
GPRAs: Glycoprotein IIb/IIIa receptor antagonists
HIT: Heparin-induced thrombocytopenia
hs-cTnT: High-sensitivity cardiac troponin T
LAD: Left anterior descending
LCX: Left circumflex artery
LM: Left main
MI: Myocardial infarction
PCI: Percutaneous coronary intervention
RCA: Right coronary artery
